# Diversity and Meta-Analysis of Microbial Differential Abundance in Nasal Metatranscriptomic Profiles of Asthma

**DOI:** 10.70322/jrbtm.2024.10018

**Published:** 2024-11-04

**Authors:** Andrew Li, Molin Yue, Xiangyu Ye, Kristina Gaietto, Anna F. Wang-Erickson, Wei Chen

**Affiliations:** 1 Department of Pediatrics, University of Pittsburgh School of Medicine, Pittsburgh, PA 15213, USA; 2 North Allegheny Senior High School, Pittsburgh, PA 15090, USA; 3 Department of Biostatistics and Health Data Science, University of Pittsburgh School of Public Health, Pittsburgh, PA 15261, USA; 4 Department of Epidemiology, University of Pittsburgh School of Public Health, Pittsburgh, PA 15261, USA

**Keywords:** Asthma, Nasal microbiome, Meta-transcriptomics, Meta-analysis, Alpha diversity, Beta diversity, Differential abundance analysis

## Abstract

Asthma affects millions worldwide and involves complex genetic, immunological, and environmental factors. The nasal microbiome is increasingly recognized for its role in asthma development, but inconsistent results and small sample sizes have limited a clear understanding. We aimed to clarify the nasal microbiome’s role in asthma using large datasets and meta-transcriptomic analysis. RNA-seq data was analyzed from two large public studies: GALA II (694 children of Puerto Rican heritage; 441 asthmatics, 253 controls) and CAAPA (562 individuals of African ancestry; 265 asthmatics, 297 controls). After quality control and host read removal, microbial reads were annotated using Kraken2. α and β diversity analyses compared microbial diversity between asthmatic and control groups. Differential abundance analysis was conducted separately, controlling for age and sex, with results combined via meta-analysis. We found that asthmatic patients exhibited significantly higher α diversity indices (Shannon, Berger-Parker, Inverse Simpson, Fisher’s) in nasal microbiota compared to controls in GALA II, with similar trends in CAAPA. β diversity analysis showed significant differences in microbial composition in GALA II data. Differential abundance analysis identified 20 species in GALA II and 9 species in CAAPA significantly associated with asthma. Meta-analysis revealed 11 species significantly associated with asthma, including *Mycobacterium_tuberculosis*. Our study demonstrates increased nasal microbiome α diversity in asthmatic patients and identifies specific microbial species associated with asthma risk. These findings enhance understanding of asthma pathogenesis from the nasal microbiome perspective and may inform future research and therapeutic strategies.

## 1. Introduction

Asthma is a heterogeneous disease characterized by chronic airway inflammation, airway hyperresponsiveness, and reversible airflow obstruction, which affects about 262 million worldwide and causes an economic burden of $80 billion in the United States alone [1,2]. The etiology of asthma is complex and far from completely understood, with genetic, immunological, and environmental factors all contributing to the development of asthma [3–6]. In recent years, the human microbiome has proven to have had a significant impact on the development of the host immune system and overall maintenance of health, offering an excellent opportunity to deepen our understanding of the mechanisms of asthma from a new perspective [7,8].

The human nasopharynx is colonized by a diverse community of commensal microbiota, which serve as “gatekeepers” for respiratory health [9]. Though limited evidence is available to date, the role of nasal microbiome in asthma has garnered increasing attention because of its inherent connection and shared biology with upper and lower airways, and paramount exposure to pathogens and bacteria. An early-life dysbiosis in the microbiota can often lead to an increased risk of developing asthma [10–13]. Based on a prospective design, McCauley et al. performed an amplicon sequencing targeting the 16S ribosomal RNA gene. They found that children’s nasal microbiome exhibited seasonal variability, which could be associated with an increased risk of asthma exacerbation [14]. However, unrobust, and even contradictory results still exist in previous studies, which may be partly due to their relatively small sample sizes. For example, Zhou et al. examined in 102 samples the association of nasal microbial diversity with the yellow zone, a period of early loss of the control of asthma, and found a significantly higher Shannon’s Index in yellow zone samples. Another study of 68 nasal brushing samples reported a decreased α diversity in asthmatics compared to healthy control samples [15,16]. Interestingly, a recent systematic review showed that, among ten studies on respiratory microbiota and asthma included, only four reported a positive association between α diversity and asthma risk, while two showed a negative association and the other four showed no significance. A meta-analysis of all 10 studies showed no significance in the differences between Shannon, Chao1, and Simpsons index [17]. In addition, using amplicon sequencing, most studies usually stopped at reporting associations at the genus or even higher levels [4,14,18]. These limitations greatly obstructed the understanding of asthma pathogenesis from the perspective of the nasal commercial microbiome and its application in management and treatment.

To address these limitations, in the current study, we utilized two published RNA-seq datasets with relatively large sample sizes, employing meta-transcriptomic analytical strategies to compare the nasal microbiome composition between asthmatic and healthy control individuals through diversity analysis and meta-analysis of two independent differential abundance analyses. This project aims to determine differences in microbial diversity between asthma and non-asthma patients and facilitate further research into an understudied yet insightful portion of the human microbiome.

## 2. Materials and Methods

### 2.1. Data Selection and Preprocessing

We analyzed data from two independent multicentered studies on the genetic factors influencing asthma. The GALA II study enrolls 4427 children of Puerto Rican heritage aged 8–21, chosen from 10 sites across both the United States and Puerto Rico [19]. NA-seq data was gathered by taking nasal airway epithelium brushings of 694 participants; 441 were characterized as “asthmatic” and 253 were characterized as “healthy control”. The second dataset is from the Consortium on Asthma among African-ancestry Populations in the Americas (CAAPA) study [20], which enrolled 14,548 individuals of all ages of African Ancestry across 15 locations. Nasal airway epithelium brushings were taken from 562 participants, 265 “asthmatic” individuals and 297 “not asthmatic” individuals (Table 1).

The raw FASTQ files from both studies (GSE152004 and GSE240567) were downloaded from the GEO platform. We then utilized the nf-core [21] tax-profiler [22] pipeline to preprocess the FASTQ files and extract the microbiome components from the samples. First, RNA-seq samples underwent quality control using fastp [23]. The sequencing data were then aligned to the human reference genome (GRCh38 [24]) with Bowtie [25] to remove the host genome components. The remaining reads were annotated and quantified using Kraken2 [26] with the Maxikraken2_1903_140GB database (March 2019), which includes archaea, bacteria, fungi, protozoa, and viral sequences from the Loman Lab [27]. Finally, we generated both an absolute abundance matrix and a relative abundance matrix for all 694 samples using Bracken for downstream analysis. Asthma status and sequencing run IDs (SRR) in the abundance matrices were matched to the samples using accession numbers (GSM) from the series matrix file as a reference.

### 2.2. Diversity Analysis

Using the derived absolute abundance matrix and relative abundance matrix, we performed α and β diversity analysis to evaluate the similarity and differences in nasal microbial community composition between asthmatic and non-asthmatic samples in each independent dataset. Four α diversity indices (Shannon, Berger-Parker, Inverse Simpsons, and Fishers), which quantify the species richness within a single sample, were calculated for each sample using KrakenTools [17,28]. A Wilcoxon rank-sum test was conducted to evaluate differences between groups. An increase in the index value indicates greater α diversity for Shannon, Inverse Simpsons, and Fishers indices. In contrast, a lower value corresponds to greater α diversity for the Berger-Parker index. For β diversity analysis, which is a comparison of microbial diversity between two groups of samples, a Bray-Curtis dissimilarity matrix using KrakenTools was first calculated, followed by a permutational multivariate analysis of variance (PERMANOVA) using the vegan (version 2.6.6.1) package for differences comparison [29]. Principal Coordinate Analysis (PcoA) was also employed to visualize the distance across samples based on the calculated dissimilarity matrix. Results with *p*-value < 0.05 were defined as statistically significant.

### 2.3. Differential Abundance Analysis

Species with a mean abundance of less than 2 and a median abundance of less than 2 across all samples were removed from the absolute abundance matrix. Then, using the DESeq2 package [30], differential abundance analysis (DAA) was first conducted separately on each independent dataset to identify taxa associated with asthma. We controlled for age and sex as covariates in the differential abundance analyses of both GALA II and CAAPA study datasets. Differentially abundant taxa (DAT) were defined as microbial taxa with Benjamini & Hochberg adjusted *p*-value < 0.05. Finally, a fixed-effects meta-analysis was conducted using the metafor package [31] (version 4.6.0) to provide a more precise estimate of DAT across studies. Data preprocessing was performed using KrakenTools in Python (version 3.9.13), and all statistical analyses were conducted in R (version 4.4.1).

## 3. Results

The baseline characteristics of the participants in both the GALA II and CAAPA study are detailed in Table 1. The GALA II study had 441 cases and 253 control samples. The median age of the case group was 13.6 years, while the control group had a mean age of 14.1 years. The proportion of females was significantly higher in the control group (57.3%) compared to the case group (49.4%) (*p* < 0.05). The CAAPA study included 265 cases and 297 controls, with mean ages of 30.0 and 29.0 years, respectively. Approximately 60% of participants in both groups were female.

**Figure 1.**
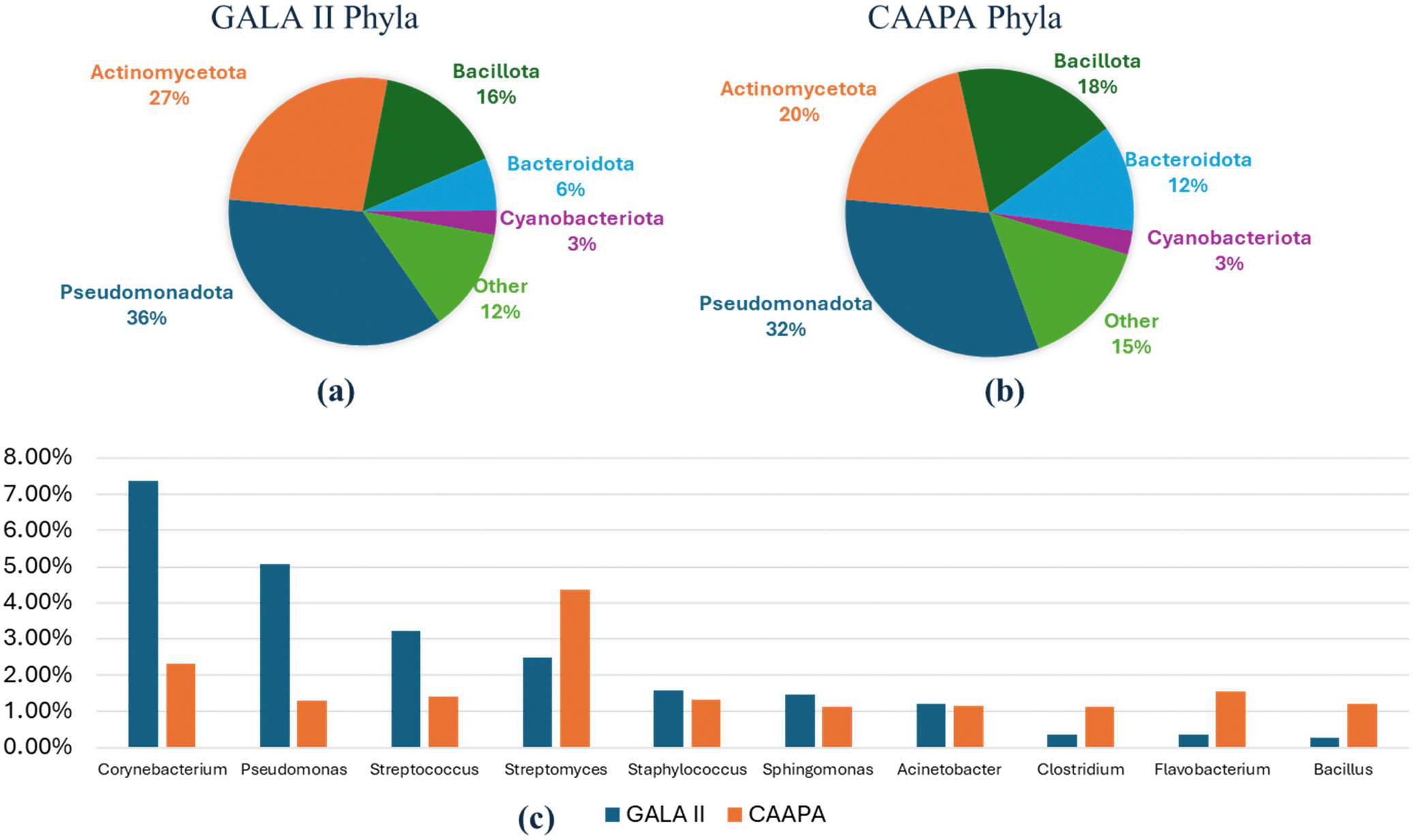
Comparison of Phyla and Genera Composition Between Study Datasets. (**a**) GALA II’s five most abundant phyla and all other individual phyla < 2%. (**b**) CAAPA’s five most abundant phyla and every other individual phylum < 2%. (**c**) Comparison of 10 most abundant genera between GALA II and CAAPA study datasets reveals a similar composition between datasets on a genus level.

A total of 1084 and 4278 unique species were detected in at least one sample in the GALA II study data and CAAPA study data, respectively. In the GALA II study data, the five species with the highest relative abundance detected were *Mycobacterium canettii* (65.49%), *Klebsiella pneumoniae* (8.88%), *Streptomyces lividans* (4.30%), *Xanthomonas euvesicatoria* (3.69%), and *Enterococcus faecium* (2.79%); in the CAAPA study data, *Mycobacterium canettii* (38.26%), *Corynebacterium segmentosum* (11.28%), *Corynebacterium glutamicum* (6.69%), *Corynebacterium propinquum* (5.96%), and *Klebsiella pneumoniae* were (5.56%) were the most prevalent species. Both datasets exhibited a similar composition, being made up predominantly of five phyla—*Pseudomonadota, Actinomycetota, Bacillota, Bacteroidota*, and *Cyanobacteriota*. These five phyla collectively accounted for over 85% of the total relative abundance in both datasets. Moreover, the relative abundances of these five phyla were closely comparable across the two datasets. In both datasets, 7 genera: *Corynebacterium, Pseudomonas, Streptococcus, Streptomyces, Staphylococcus, Sphingomonas*, and *Acinetobacter* had a relative abundance greater than 1%, with each genus ranking among the top 10 most abundant genera (Figure 1).

**Figure 2.**
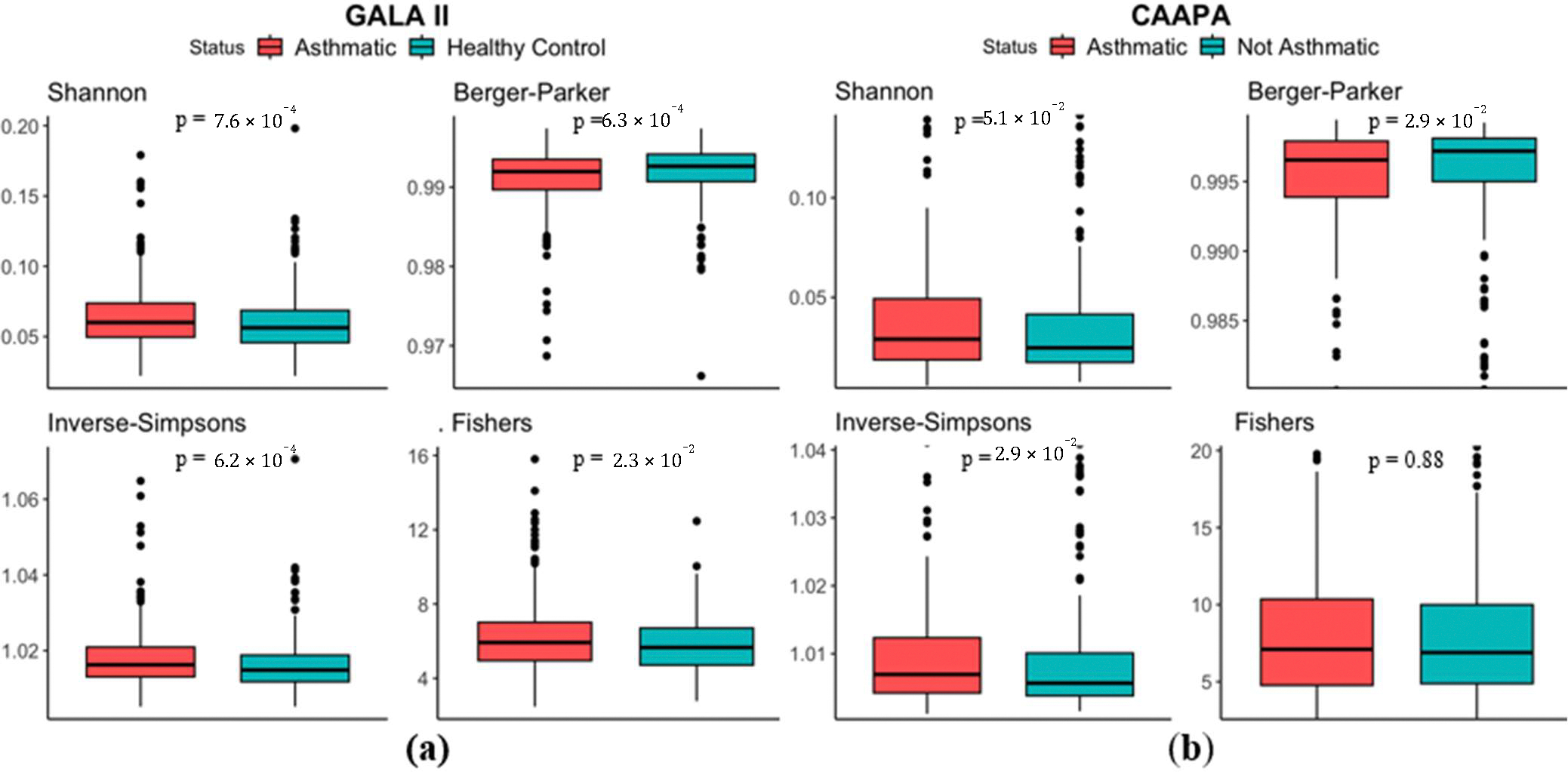
α Diversity comparison between asthmatic and non-asthmatic individuals using Shannon, Berger-Parker, Inverse-Simpsons, and Fishers Indices. (**a**) In the GALA II study dataset, all four indices indicate greater α diversity among asthmatics and significant differences based on Wilcoxon test. (**b**) In the CAAPA study dataset, all four indices indicate greater α diversity among asthmatics, but only significant differences in the Berger Parker and Inverse-Simpsons indices.

Analysis based on the 694 Hispanic children of the GALA II study showed significant higher Shannon Index (median in asthmatics (*Mdn*_A_) = 0.0600, median in healthy controls (*Mdn*_HC_) = 0.0562, *p* < 0.001), Inverse Simpsons Index (*Mdn*_A_ = 1.02, *Mdn*_HC_ = 1.01, *p* < 0.001), Fishers index (*Mdn*_A_ = 5.92, *Mdn*_HC_ = 5.66, *p* = 2.30 × 10^−2^), and lower Berger Parker Index (*Mdn*_A_ = 0.992, *Mdn*_HC_ = 0.993, *p* < 0.001) in samples from the asthmatic group, indicating an increased microbial α diversity in the nasal cavity of asthmatics (Figure 2a). Results from the 562 CAAPA Participants of African descent further supports this finding, with Berger Parker Index (*Mdn*_A_ = 0.996, *Mdn*_HC_ = 0.997, *p* = 2.90 × 10^−2^) and Inverse Simpsons Index (*Mdn*_A_ = 1.007, *Mdn*_HC_ = 1.006, *p* = 2.90 × 10^−2^) reproducing significant differences, although Shannon Index (*Mdn*_A_ = 0.0290, *Mdn*_HC_ = 0.0246, *p* = 5.10 × 10^−2^) and Fisher’s Index (*Mdn*_A_ = 7.10, *Mdn*_HC_ = 6.89, *p* = 0.880) presented no statistical significance (Figure 2b).

**Figure 3.**
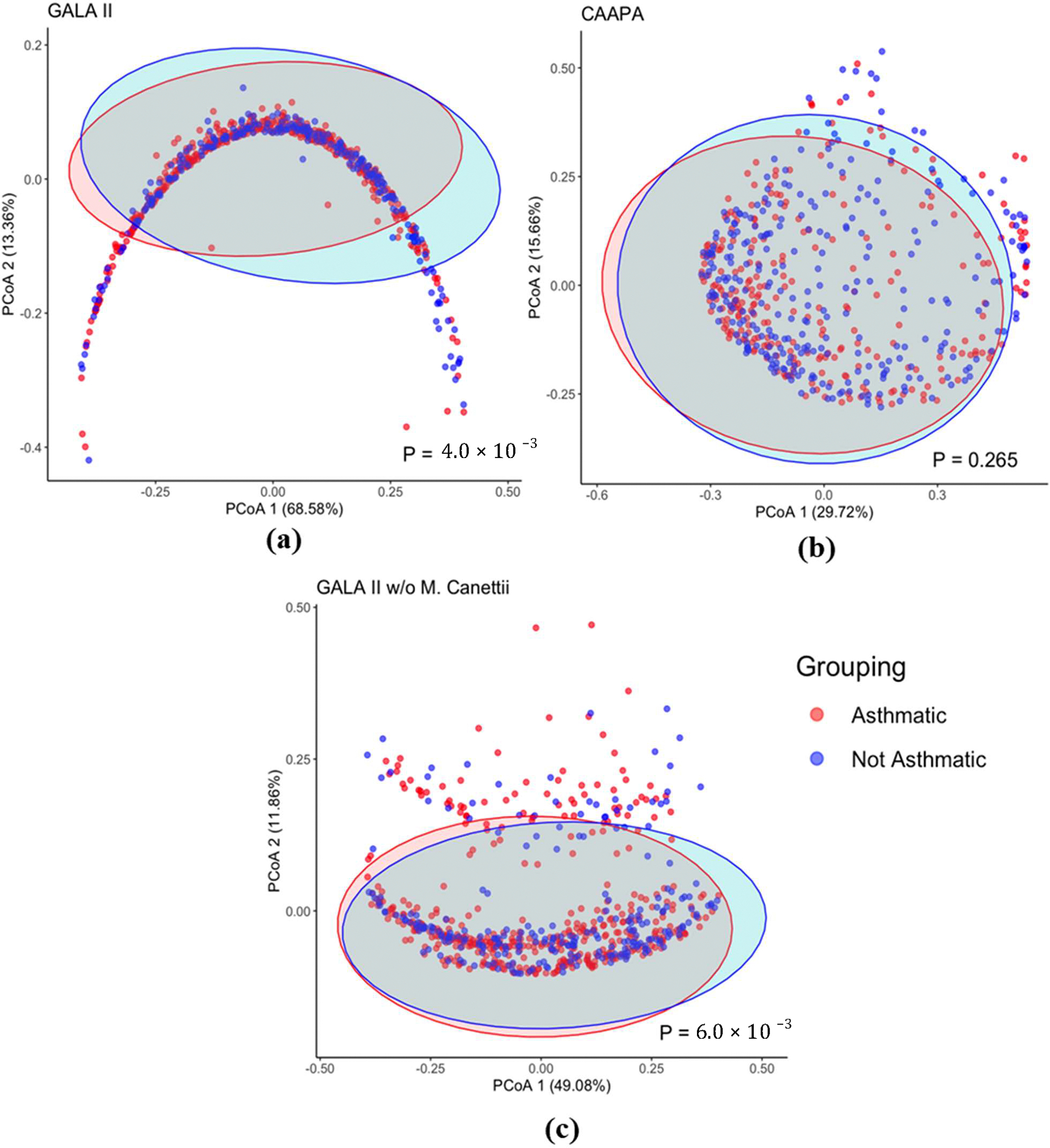
β Diversity Comparison Between Asthmatic and Non-asthmatic Individuals. (**a**) The PCoA plot of the GALA II Bray Curtis dissimilarity matrix shows a significant difference between the composition of groups. (**b**) PCoA plot using CAAPA Bray Curtis dissimilarity matrix reveals no significant difference in composition between groups. (**c**) Recalculated PCoA plot of the GALA II study dataset after removing the most abundant species *Mycobacterium Canettii* (65%); the horseshoe shape is no longer present and maintains a significant difference in microbial composition between groups.

Based on the Bray-Curtis dissimilarity matrix, we also found a significant difference in overall microbiome composition between asthmatics and healthy control patients from GALA II study data (*p* = 4.00 × 10^−3^), which is not observed, however, in nasal samples from the CAAPA study (*p* = 0.265) (Figure 3).

**Figure 4.**
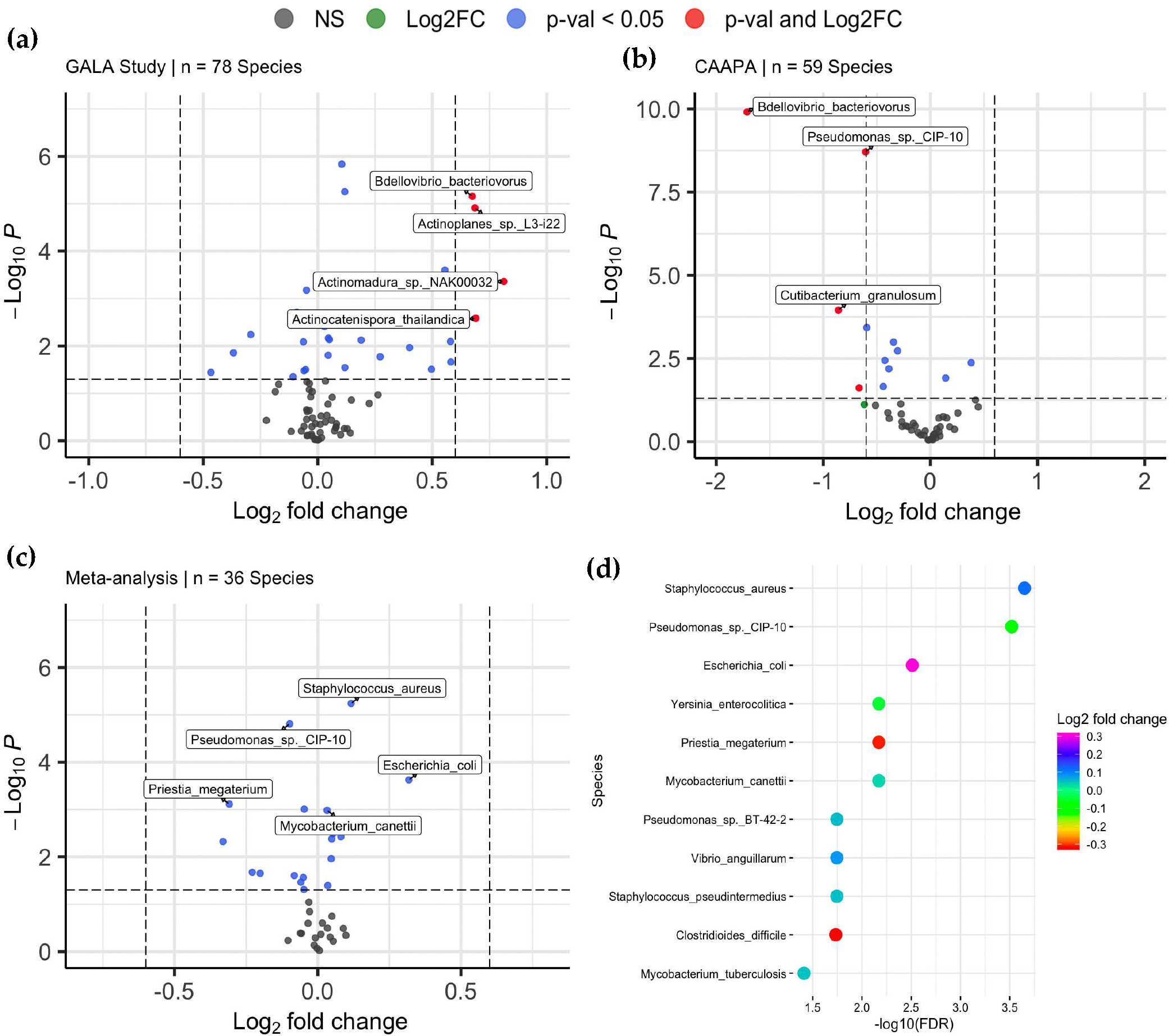
Differentially Abundant Taxa between Asthmatics and Non-asthmatics. (**a**) Volcano plots of 78 species were included in the DAA of the GALA II study dataset, of which 20 were associated with asthma risk. (**b**) Volcano plots of 59 species were included in the DAA of the CAAPA study dataset, of which 9 species were found to be associated with asthma risk. (**c**) Volcano plots of 36 species was included in meta-analysis, where 11 species were associated with asthma. (**d**) The plot shows the relationship between the log_2_-fold change and *p*-value of each species associated with asthma in the meta-analysis.

With species of low abundance excluded (mean abundance < 2 and median abundance < 2, Materials and Methods), 78 and 59 species were included in DAA for the GALA II study data and CAAPA study data, respectively. For the GALA II study data, a total of 20 species, such as *Chlorobaculum parvum* (log_2_FC = 0.105, *P*_*adj*_ = 1.14 × 10^−4^), *Staphylococcus pseudintermedius* (log_2_FC = 0.0466, *P*_*adj*_ = 3.35 × 10^−2^), *Staphylococcus aureus* (log_2_FC = 0.118, *P*_*adj*_ = 1.80 × 10^−4^), *Bdellovibrio bacteriovorus* (log_2_FC = 0.675, *P*_*adj*_ = 1.80 × 10^−4^), and *Actinomadura* sp. *NAK00032* (log_2_FC = 0.812, *P*_*adj*_ = 4.86 × 10^−3^) showed a significant difference in abundance between samples from asthmatic and healthy control individuals, with five species negatively enriched and 15 species positively enriched in the asthmatic group compared to the healthy controls. (Figure 4a and Table S1). Of note, 16 of the 20 species associated with asthma are classified under 2 phyla: *Actinomycetota* or *Psudomonadota*. For the CAAPA study data, 9 species showed significant differential abundance, and only one species was positively enriched in the asthmatic group compared to healthy control, including *Bdellovibrio bacteriovorus* (log_2_FC = −1.71, *P*_*adj*_ = 5.87 × 10^−9^), *Pseudomonas* sp. *CIP-10* (log_2_FC = −0.605, *P*_*adj*_ = 4.66 × 10^−8^), *Cutibacterium granulosum* (log_2_FC = −0.859, *P*_*adj*_ = 1.78 × 10^−3^), *Clostridium botulinum* (log_2_FC = −0.345, *P*_*adj*_ = 9.80 × 10^−3^), and *Priestia megaterium* (log_2_FC = −0.595, *P*_*adj*_ = 4.46 × 10^−3^) (Figure 4b and Table S2).

Meta-analysis of the two studies was conducted on the 36 species that were common to the differential abundance analysis models of both studies. 11 species, such as *Staphylococcus aureus* (log_2_FC = 0.117, *P*_*adj*_ = 2.24 × 10^−4^), *Mycobacterium canettii* (log_2_FC = 0.0326, *P*_*adj*_ = 6.75 × 10^−3^), and *Escherichia coli* (log_2_FC = 0.318, *P*_*adj*_ = 3.09 × 10^−3^), showed significant differential abundance between asthmatic and healthy control groups, among which 7 were positively enriched in asthmatic samples. Of note, *Pseudomonas* sp. *CIP-10*, which presented as DAT in both individual analyses, also showed significance in meta-analyses, indicating its robustness across datasets. Interestingly, *Mycobacterium tuberculosis* (log_2_FC = 0.0470, *P*_*adj*_ = 3.88 × 10^−2^) and *Clostridioides_difficile* (log_2_FC = −0.331, *P*_*adj*_ = 1.84 × 10^−2^) were species significant only in the meta-analysis, while 16 species with significance in either GALA II study data or CAAPA study data showed no statistical significance in meta-analysis, including *Chlorobaculum parvum*, *Actinoplanes* sp. *L3-i22, Bdellovibrio bacteriovorus, Streptomyces lydicus, Cutibacterium_acnes*, and *Actinomadura*_sp._*NAK00032* (Figure 4c,d, and Table S3).

## 4. Discussion

In our study, we conducted a diversity analysis and differential abundance meta-analysis of two independent datasets, GALA II and CAAPA, which revealed a greater intrasample diversity within asthmatics compared to non-asthmatic patients and identified 22 species significantly associated with asthma. However, there was no indication of a difference in intersample diversity between groups.

The true relationship between the nasal microbiome and asthma is still unclear, partly due to the limited sample size of previous studies. However, our results are consistent with multiple previous studies that reported higher α nasal microbial diversity in asthma patients. For example, Zhou et al. examined in 102 samples the association of nasal microbial diversity with the yellow zone (a period of early loss of the control of asthma). They found a significantly higher Shannon’s Index in yellow zone samples [16]. In another study of nasal brushings from 72 participants, α diversity measured by Faith’s phylogenic diversity index, which focuses on the richness of evolutionary lineages, indicated a positive, but not statistically significant, trend between nasal bacterial α diversity and asthma activity [32]. With a much larger sample size, our results extended these findings to four other indices, including Shannon, Berger-Parker, Simpsons, and Fishers, which are more robust and reliable.

Interestingly, even though the PERMANOVA test (*p* = 4.00 × 10^−3^) for β diversity in the GALA II study data suggested a significant difference between groups, the PcoA plot presented a horseshoe-like shape lacking distinct clustering of groups. This indicates the presence of the horseshoe effect, a common limitation potentially resulting from the extreme variability in the abundance data. It is most often observed in dimension-reducing strategies, resulting from an inability to discriminate among samples that don’t share common features [33]. In a distance matrix, while Euclidean distance typically increases linearly with the gradient, it often becomes saturated once it surpasses a certain threshold. At extreme values, even when substantial dissimilarity exists between samples, the Euclidean distance metric may represent them as equally dissimilar [33]. As we recalculated the distance matrix with the top species *Mycobacterium canettii* excluded, which had originally accounted for on average a disproportionate 64% of the total species abundance within a sample, the PcoA plot no longer shows indication of the horseshoe effect, while PERMANOVA still revealed a significantly different β diversity between groups (*p* = 6.00 × 10^−3^) (Figure 3c).

Equally important, we also revealed taxa associated with asthma at the species level, among which several have never been reported before. For example, *Pseudomonas* sp. *CIP-10* showed significant enrichment in both individual DAAs and the combined meta-analysis DAA. To date, little is known about its role in physiology and disease. On the other hand, *Pseudomonas, p. aeruginosa*, another species in its genus level, has been well documented for its role in chronic lung infections, including asthma [34], where it may induce inflammation and contribute to microbial dysbiosis in the lungs [35]. Our meta-analysis also identified *Mycobacterium canettii* and *Mycobacterium tuberculosis* as species associated with asthma. Both species belong to the *Mycobacterium tuberculosis* Complex, a group of closely related bacteria that can act as opportunistic pathogens for tuberculosis in humans and animals [36,37]. Other members of this complex include *M. bovis, M. microti, M. africanum, M. pinnipedii*, and *M. caprae*. Other “nontuberculous” *Mycobacterium* species (NTM) are increasingly recognized for their role in human disease, such as *M. leprae*, which is known to cause leprosy in humans. These NTMs have also been linked to asthma. A study indicates that NTM infections occur nearly 100 times more frequently in asthmatic populations (1.7%) compared to the general population (0.017%). Given the significant role *Mycobacterium tuberculosis* complex species already play in the development of diseases like tuberculosis, as well as the association between NTM species and asthma development, *M. tuberculosis* and *M. canettii* may exhibit similar pathogenic behavior in relation to asthma. However, further research is needed to confirm this potential linkage and elucidate the underlying mechanisms [38].

In addition, some other notable species from the *Staphylococcus* genus, specifically *S. aureus, S. homonis* and *S. pseudintermedius*, are known opportunistic pathogens of asthma [39–41]. For example, *S. aureus* infections often induce or intensify inflammatory responses, contributing significantly to the development of allergic asthma [42]. Notably, species from the *Moraxella* and *Haemophilus* genera, though previously linked to asthma, were not found to be associated with asthma risk in our study [43–47]. Future research could benefit from additional analyses, such as functional pathway analysis, to better understand the mechanisms underlying the role of these species. In summary, our research highlights a greater microbial diversity among asthmatic patients. It identifies established and emerging microbial species linked to asthma, underscoring the need for further investigation and refinement in this field.

However, some limitations still exist within our study. Firstly, while α and β diversity measures can be used as possible indicators for early asthma detection by measuring microbiota dysbiosis during infancy or as a guide for tailoring antibiotic treatments aimed at reducing microbial diversity, the clinical significance of our results remains limited [47]. This is because of the small differences in median alpha diversity between groups, making a clear interpretation in clinical settings challenging to distinguish, as well as the lack of robustness across beta diversity findings. Second, our analysis focused exclusively on the presence of asthma. Given that previous research has highlighted the importance and greater heterogeneity of asthma endotypes, future studies should explore the associations between the nasal microbiome and different asthma endotypes [48]. Additionally, while we obtained results consistent with previous studies from two relatively large datasets, both were case-control studies, which may offer limited insights into the relationship between the nasal microbiome and asthma. A prospective study design would help clarify the causal links between specific microbial species and asthma development. Additionally, relying solely on meta-transcriptomics data leaves the mechanisms behind these associations unclear; integrating tools like metabolic analysis could help uncover the potential functional basis.

## 5. Conclusions

In summary, by employing datasets of larger sample sizes and meta-transcriptomic analytical strategies, our study provides greater insight into the role of the nasal microbiome in asthma risk. Diversity analysis reveals a greater α diversity within asthmatic patients as compared to healthy control patients but no difference in β diversity between groups. Meta-analysis of two individual differential abundance analysis identifies 20 species associated with asthma risk. Our findings will hopefully facilitate further meta-transcriptomic research in the nasal microbiome, specifically exploring the species associated with asthma that have not been widely studied or reported.

## Supplementary Material

Supplementary Information

## Figures and Tables

**Table 1.** Baseline characteristics of patients in GALA II and CAAPA study datasets.

	GALA II			CAAPA		
	Case (n = 441)	Control (n = 253)	*p*-Value	Case (n = 265)	Control (n = 297)	*p*-Value
Demographics						
Age	13.6 (11.4–16.4)	14.1 (11.6–16.5)	0.686	30.0 (16.0–45.0)	29.0 (16.0–44.0)	0.769
Female Sex	218 (49.4)	145 (57.3)	0.045	161 (60.8)	181 (60.9)	0.964

Data are presented as n (%) or median (interquartile range). GALA II participants are of Puerto Rican heritage and are spread across North America; CAAPA participants are of African ancestry and live in the United States.
